# Pathogen dematerialization and the ABS loophole^1^

**DOI:** 10.1093/jlb/lsad002

**Published:** 2023-03-04

**Authors:** Abbie-Rose Hampton

**Affiliations:** King's College London, London, UK

**Keywords:** Access and Benefit-Sharing, Equity, Global Health Justice, International Law, Pandemic Preparedness, Pathogen Dematerialization

## I. INTRODUCTION

As COVID-19 has most recently highlighted, the timely sharing of pathogen samples (both their physical form and associated information) plays a crucial role in preparing for and responding to health emergencies.^2^ The level of importance attributed to pathogen sharing is a direct result of access to novel pathogen samples not only facilitating the completion of risk assessments but also permitting the adaptation of anti-viral medication and the development of vaccine and diagnostic kits,^3^ all of which are vital for the protection of global public health.^4^ Serving as a somewhat reflection of this importance, pathogen samples have traditionally been treated as ‘common pool resources’, which third-party users were free to access when necessary and which were shared informally on a global scale.^5^

The ‘soft global norm’^6^ of informally sharing pathogen samples has been called into question, however, following the 2007 H5N1 influenza pandemic and the decision of the Indonesian Government to assert sovereignty^7^ over their genetic resources,^8^ withholding access to H5N1 samples which had been collected within their territorial borders in the process.^9^ The deliberate break away from a long-established global norm by the Indonesian Government here was in response to their belief—and that of many developing states—that the pathogen sharing system was inequitable and unfair; a system that expected developing states to provide free and timely access to pathogen samples collected within their borders but failed to ensure that these states would then be able to access the benefits—such as vaccine and anti-viral medication—which result from their utilization.^10^ The deliberate actions of the Indonesian Government here shed light on two distinct issues which exist, and have done throughout history, within global public health. These issues can be summarized as follows:

- The need for researchers and other actors within the field of pandemic preparedness to gain access to novel pathogen samples in a timely manner; and
- The limited availability of vaccine and other medical countermeasures during a health emergency, with developing states typically struggling to gain sufficient and timely access to such resources due to developed states dominating procurement.^11^

Developing states have long faced considerable struggles to procure sufficient access to medical countermeasures during health emergencies.^12^ The experiences of the Global South during the COVID-19 pandemic have most recently highlighted the overwhelming disparities which exist between developed and developing states during a health emergency;^13^ with this presenting a problem for global public health and global health justice. The favored solution to these problems has been the replacement of the informal system of pathogen sharing with one which uses transactional Access and Benefit Sharing (ABS) agreements; with developing states now being encouraged to use their sovereign genetic resources,^14^ in this case pathogen samples, as ‘bargaining chips’ which can be traded in exchange for access to medical countermeasures during a health emergency.^15^ With the intention of placing the equitable sharing of benefits on equal footing with the sharing of pathogen samples, the use of ABS within global public health seeks to ensure the provision of equity, fairness and justice in relation to the ability of developing states to access medical countermeasures during a health emergency, whilst also ensuring the timely sharing of pathogen samples on a global scale.

The ability of ABS agreements within global public health to achieve equity and fairness in the distribution of benefits has become increasingly threatened in recent years by rising levels of pathogen dematerialization; with pathogen dematerialization referring to the act(s) of separating information from physical pathogen samples, thereby reducing the need to access physical samples. This paper presents the argument that the ability of dematerialized pathogen samples to circumvent the benefit-sharing obligations of ABS mechanisms and the subsequent production of a ‘benefit-sharing loophole’ produces a number of concerns surrounding not only the functionality of ABS agreements, but also their ability to ensure fairness, equity, and global health justice. Whilst this paper initially seeks to provide a solution to the problem through identifying the avenues through which DSI could become included within the ABS transaction, it ultimately concludes that the use of ABS within global public health should be abandoned and that an alternative solution(s) must be found in order to deliver global health justice. As such, this paper will first explore the extent to which the dematerialization of pathogen samples represents a threat to global health justice, considering the use of ABS within global public health alongside the failure of international ABS agreements to include dematerialized virus samples within their scope and the impact had on the distribution of benefits as a result. Following the initial framing of the problem, this paper will conclude with an examination of the potential solutions to the myriad of problems posed by synthetic biology^16^ in the context of ABS agreements and the distribution of medical countermeasures before ultimately proposing an alternative solution.

## II. ABS IN GLOBAL PUBLIC HEALTH

Following the recognition that pathogen samples amount to sovereign genetic resources, as opposed to ‘common pool resources’,^17^ the use of ABS has expanded beyond its original role in biodiversity conservation^18^ to become the favored policy approach to pathogen sharing within global public health. In doing so, it has sought to provide a single ‘market-based solution’ to the two distinct public health problems outlined above: the need for third party users to gain timely access to pathogen samples and the difficulties faced by developing states with regards to accessing medical countermeasures during a health emergency.^19^ Taking the form of a transaction between the ‘user’ and ‘provider’ of genetic resources, the ABS agreement regulates the way in which genetic resources can be accessed and how the benefits resulting from their utilization should then be shared with the ‘provider’.^20^ A trade occurs between the two parties, with access to genetic resources being contingent upon access to benefits and vice versa.

In the context of pathogen sharing, developing states trade or provide access to the physical pathogen samples collected within their borders in exchange for access to vaccine and other medical countermeasures during a health emergency. These ABS obligations are provided by the Convention on Biological Diversity^21^ (CBD or the Convention); its supplementary agreement, the Nagoya Protocol on Access to Genetic Resources and the Fair and Equitable Sharing of Benefits Arising from their Utilization to the Convention on Biological Diversity^22^ (Nagoya Protocol); and the Pandemic Influenza Preparedness Framework^23^ (PIP Framework).^24^

### II.A. The CBD & the Nagoya Protocol

The ABS obligations provided for by the CBD and the Nagoya Protocol find their basis in the mutual aim of both agreements to ensure ‘the fair and equitable sharing of the benefits arising out of the utilization of genetic resources’.^25^ Both the CBD and the Nagoya Protocol favor a bilateral approach to the ABS transaction, with such agreements being negotiated between two parties—the ‘user’ and the ‘provider’ of genetic resources. Building upon the ABS obligations first specified by the CBD,^26^ the Nagoya Protocol places an obligation on ‘users’ of genetic resources to first obtain Prior Informed Consent (PIC),^27^ with this taking the form of a permit which allows the ‘user’ to collect genetic resources from a specified area.^28^ In addition to PIC, Mutually Agreed Terms (MAT) must also be negotiated,^29^ with these terms specifying not only the way in which those resources may then be utilized, but also the benefit-sharing obligations which the ‘user’ is required to agree to before access to genetic resources can be granted.^30^

Whilst the use of MAT by the CBD and the Nagoya Protocol offers a level of assurance to ‘providers’ of genetic resources that benefits will be shared, with it being the use of MAT here which makes the ABS agreement a legally binding contract, issues surrounding compliance with national ABS legislation and the ABS negotiation process, specifically the length of negotiations,^31^ cast doubt on the suitability of bilateral ABS agreements to pathogen sharing—where access to samples is time sensitive—and on their ability to procure sufficient benefits for developing states during a health emergency.

### II.B. The PIP Framework

The PIP Framework seeks to create ‘a fair, transparent, equitable, efficient, effective system for, on an equal footing: (i) the sharing of H5N1 and other influenza viruses with human pandemic potential and (ii) access to vaccines and sharing of other benefits’.^32^ Restricted in scope to the sharing of influenza viruses with human pandemic potential and the benefits resulting from their use,^33^ the Framework takes a multilateral approach to the ABS transaction, with the World Health Organization (WHO) taking on the role of ‘mediator’ as opposed to negotiations simply taking place between the ‘user’ and ‘provider’ of samples.^34^ The role of the WHO here as ‘mediator’ was expected to result in a more just and fair outcome for developing states.^35^ Doubt has been cast as to whether these expectations have and are capable of truly being met, however, with the bargaining power of the WHO as an intermediary actor here lacking the strength and conviction which was anticipated during the negotiation of the PIP Framework.

The Framework provides for recommendations in two areas: the timely sharing of influenza samples with human pandemic potential between member states and the WHO via GISRS;^36^ and the sharing of virus samples with third-party entities that operate outside of the GISRS network, such as vaccine manufacturers, in return for these external entities sharing benefits with the WHO for distribution to member states.^37^ The PIP Framework introduced ABS through the use of legally binding Standard Material Transfer Agreements (SMTAs),^38^ with their standard frameworks changing in accordance with whether the sample is being shared with a GISRS or non-GISRS entity.^39^ Whilst SMTA1s refer to the transfer of samples within the GISRS Network, SMTA2s place benefit-sharing obligations on those Non-GISRS entities which receive access to influenza viruses samples with human pandemic potential.^40^ In order for a non-GISRS entity to receive access to virus samples, they must enter into a SMTA2 and commit to a minimum of two of the benefits specified by Annex 2 of the PIP Framework.^41^ Although the use of SMTA2s by the PIP Framework was expected to ensure the fair and equitable distribution of pandemic influenza vaccine and other medical countermeasures during an influenza pandemic,^42^ the WHO has consistently being unable to secure sufficient benefit-sharing commitments from vaccine manufacturers and other members of the pharmaceutical industry.^43^ With the PIP Framework yet to be put to the test during an influenza pandemic, it appears unlikely that it will work as expected with regards to its ability to deliver sufficient benefits—alongside equity, fairness, and justice—to developing states.

### II.C. ABS: A Great Success?

Whilst the form which ABS takes may differ across the CBD, the Nagoya Protocol and the PIP Framework, the approach remains transactional in nature.^44^ Although taking such an approach comes with an array of problems, largely due to the conflicting interests of each party to the transaction,^45^ the use of ABS as a whole has been praised as a means of delivering justice, equity and fairness to developing states.^46^ Much of this praise has translated to its use in global health amidst claims that the PIP Framework’s use of ABS was its ‘greatest accomplishment for equity’.^47^ Such praise is to be somewhat expected given the rationale behind the use of ABS more broadly and its supposed role in reducing colonial inequalities and rectifying the power imbalances which have long existed between developed and developing states as a result of biopiracy^48^ and bio-colonialism.^49^ Emerging from the demands of the Global South, support for the use of ABS, within global public health and more broadly, flows not only from its perceived ability to place benefit-sharing on equal footing with pathogen sharing, but also the expectation that it is able to place developing states on equal footing with their developed counterparts.^50^ It is important to note at this stage, however, that much of the praise surrounding the use of ABS in global health as a means of delivering justice, equity, and fairness to developing states—whether that be specific to the context of pandemic influenza and the PIP Framework or with regards to the sharing of pathogens as a whole—is rhetorical, and premised on how it is expected to work as opposed to the actual, tangible benefits which have been delivered to developing states as a result, with expectation and reality often being misaligned here.

Despite this, ABS agreements are commonly viewed, whether rightly or wrongly, to be vital for developing states which have struggled to access vaccine and other medical countermeasures during a health emergency. With the continued existence of barriers to self-procurement, such as financing and manufacturing capacity,^51^ the successful negotiation of an ABS agreement is widely considered to be the only means of ensuring equitable access to benefits for developing states.^52^ The ability of ABS agreements to ensure equity and justice in the context of benefit-sharing, however, is increasingly under threat as a result of developments in synthetic biology and rapid levels of pathogen dematerialization.

## III. PATHOGEN DEMATERIALIZATION: A THREAT TO ABS AND GLOBAL HEALTH JUSTICE

The rapid levels of growth and development within the field of synthetic biology in recent years have undoubtedly produced an array of benefits,^53^ and potentially catastrophic risks. Until fairly recently, it was impossible to detach physical pathogen samples from the information they contained—the sample *was* the information—but technological advancements have allowed for the dematerialization of pathogen samples^54^ to occur globally. Despite Digital Sequence Information^55^ (DSI)^56^ repositories having existed since the 1990s, recent technological innovations are beginning to enable the use of this information in many areas of pandemic preparedness and response; going as far as to form a substitution for ‘physical pathogen samples during pandemic risk assessment and the development of commercial products’.^57^

Whilst physical pathogen samples continue to be of importance with regards to the testing of many medical countermeasures, including vaccine and anti-viral medication,^58^ technology will continue to advance and the importance and role of DSI in the context of global public health will grow alongside it. With researchers able to freely access and share DSI via online gene-banks, such as GenBank and GISAID, it is only a matter of time and increased technical capabilities^59^ before DSI can be used in place of physical organisms in research and development,^60^ reducing the need to negotiate access to physical pathogen samples in the process. Already, we are seeing the development of novel approaches to vaccine development which do not require access to physical pathogen samples but instead utilize DSI, with these approaches being expected to not only result in the development of better vaccine, but also to significantly decrease the time required to manufacture them.^61^ The growing role of DSI in global public health has been evidenced by the COVID-19 pandemic, where the decision of researchers to share the SARS-CoV-2 genome sequences with the rest of the world via GISAID and GenBank enabled vital research and the development medical countermeasures to begin before physical pathogen samples could be isolated and accessed.^62^

### III.A. DSI as a Threat to Global Health Justice

As the importance of DSI during the beginning of the COVID-19 pandemic has highlighted, enhanced technological capabilities with regards to the dematerialization, and potential subsequent re-materialization, of pathogen samples have the potential to provide great benefits to global public health, whether that be in the form of enhanced access to vital information or improvements in the accuracy of diagnostic tools, for example. With such capabilities being expected to develop further, it is anticipated that the dematerialization and subsequent re-materialization of pathogen samples will ‘only increase in efficiency and ease, and decrease in entry costs, leading to a proliferation of its availability’,^63^ reducing the need for access to physical pathogen samples.

The array of potential benefits arising from advances in synthetic biology are coupled with numerous risks and the potential to cause the serious loss of wellbeing and life on a global scale. Whilst the risks associated with synthetic biology are typically framed in relation to threats posed to global health security, due to an enhanced risk of bioterrorism and synthetically derived pandemics, much less consideration has been afforded to the threat synthetic biology, specifically the dematerialization of virus samples, poses to global health justice with regards to the distribution benefits, such as vaccine and other medical countermeasures.

As has already been acknowledged, the preferred solution to the difficulties faced by developing states in accessing medical countermeasures during a health emergency is the use of transactional ABS agreements which are provided for by the CBD, the Nagoya Protocol, and the PIP Framework. With DSI now beginning to assume a similar role to that of physical pathogen samples, if DSI is capable of being shared freely without the imposition of benefit-sharing obligations linked to its utilization, the attempts made by the CBD, the Nagoya Protocol, and the PIP Framework to place fair and equitable benefit-sharing on equal footing with pathogen sharing will be undermined. As such, any international ABS framework, and the manner in which that framework is incorporated into national ABS legislation, will need to reflect the view that ‘Benefit-sharing arrangements for commercial and non-commercial use of DSI should reflect the same or similar benefit-sharing obligations as those attached to biological materials’.^64^ The failure to incorporate DSI within the ABS transaction would ultimately reduce the ability of developing states to access medical countermeasures during a pandemic and could have detrimental implications for existing ABS arrangements,^65^ posing a threat to global health equity and justice in the process.

### III.B. The Benefit-Sharing Loophole

The growing use of DSI is the latest cause for dispute with regards to the negotiation of ABS agreements, most notably in relation to benefit-sharing obligations.^66^ As increased use of DSI gradually negates the need to access physical pathogen samples, questions have been raised with regards to what this means for ABS governance—are the CBD, the Nagoya Protocol, and the PIP Framework flexible enough to encompass these changes or is it the equivalent of ‘regulat(ing) VCR technology in the era of You-Tube’?^67^ Are these rapid changes in the field of synthetic biology significant enough to completely de-stabilize ABS, specifically the benefit-sharing obligations upon which developing states have become reliant in order to gain access to medical countermeasures and other benefits?^68^ With genetic resources having long been understood by their physical materiality,^69^ the application of the CBD, the Nagoya Protocol, and the PIP Framework to DSI is heavily disputed and remains unclear.^70^ The source of this uncertainty finds its origins in the scope of each legal instrument, with none of them containing explicit reference to DSI.

The CBD—and subsequently the Nagoya Protocol—defines ‘genetic resources’ as ‘genetic material of actual or potential value;’ with ‘any material of plant, animal, microbial or other origin containing functional units of heredity’ being categorized as ‘genetic material’.^71^ The reference to ‘material’ here suggests that both the CBD and Nagoya Protocol apply only physical pathogen samples, excluding ‘intangible aspects’, such as DSI, from their scope of application in the process.^72^ As DSI is broadly understood to fall beyond the scope of both instruments,^73^ neither PIC nor MAT are required, meaning that DSI can successfully be accessed under the CBD and the Nagoya Protocol without the successful negotiation of an ABS agreement and the imposition of benefit-sharing obligations.

Similarly, the primary element of the PIP Framework’s provisions on ABS—the SMTA2s—apply only to PIP Biological Materials (BM), excluding the associated DSI from their scope.^74^ Unsurprisingly, given the limited application of the PIP Framework, PIP BM only includes, ‘human clinical specimens, virus isolates of wild type human H5N1 and other influenza viruses with human pandemic potential; and modified viruses prepared from H5N1 and/or other influenza viruses with human pandemic potential developed by WHO GISRS laboratories” and “RNA extracted from wild-type H5N1 and other human influenza viruses with human pandemic potential and cDNA that encompass the entire coding region of one or more viral genes’.^75^ The exclusion of DSI from this definition means that SMTA2s are only required for access to physical samples of PIP BM and not their associated DSI.

A clear distinction exists, therefore, between the physical pathogen samples, which form part of the ABS transaction and general obligations which encourage the free and timely ‘disclosure and exchange of information about these resources’.^76^ The problem which arises here is a direct result of DSI being able to bypass the ABS obligations of the CBD, the Nagoya Protocol, and the PIP Framework, with third-party users being able to access DSI without the negotiation of an ABS agreement.^77^ This, in turn, functions to produce what is commonly referred to as a ‘benefit-sharing loophole’, where users are able to access and utilize DSI without being bound by the terms of access and benefit-sharing obligations, which stem from the use of MTAs and SMTA2s within the ABS agreement.^78^

The threat posed by the existence of a ‘benefit-sharing loophole’ here is somewhat negated in the context of the PIP Framework as ‘all current major influenza vaccine manufacturers have signed SMTA2s with WHO’, with these agreements remaining valid and enforceable regardless of whether manufacturers utilize physical pathogen samples or rely entirely on DSI in the development of vaccine in the future.^79^ There remains a risk, however, that new and emerging manufacturers will one day be able to be wholly reliant upon the use of DSI, negating the need for access to physical pathogen samples at any stage in the development process and therefore no longer requiring the negotiation of an SMTA2.

With it becoming increasingly possible for vaccine and other medical countermeasures to be developed and manufactured through the use of DSI which is freely available, the incentive for entering into an ABS agreement is significantly diminished.^80^ Concerns surrounding the ability of DSI to bypass ABS and the risk that researchers may opt to synthesize genetic resources rather than be subject to benefit-sharing obligations and access restrictions^81^ are met with equally pressing concerns surrounding the detrimental impact that including DSI within the ABS transaction may have on ‘information exchange and open science’, both of which are vital for a timely response during a health emergency.^82^ It is questionable as to whether ABS agreements, such as those provided for by the CBD, the Nagoya Protocol, and the PIP Framework, are capable of keeping up with scientific advances in relation to DSI or even if they are the most suitable means of regulating access to DSI and the sharing of benefits resulting from its use.^83^

The problem and how it might be addressed is the subject of ongoing discussion. Whilst recommendations for DSI to be treated in the same way as physical virus samples under the PIP Framework were made during a review in 2016,^84^ and although DSI formed a significant part of discussions at the 2016 UN Biodiversity Conference (COP13),^85^ a solution which ensures fairness, equity and justice has yet to be found.

## IV. CLOSING THE BENEFIT-SHARING LOOPHOLE

The ‘benefit-sharing loophole’ which is produced due to the ability of DSI to bypass ABS obligations functions to seriously undermine ‘global efforts to more equitably distribute the benefits of research and development’^86^ and as such, must be closed. Whilst difficulties have arisen in relation to finding a solution at the policy-level, numerous solutions for how to close the ‘benefit-sharing loophole’ have been proposed at the academic level. The remainder of this paper will examine some of these proposals in detail in order to determine not just their viability, but their ability to deliver equity and justice in global health.

### IV.A. The Role of National ABS

Whilst limited in nature to the ABS obligations under the CBD and the Nagoya Protocol, a partial solution to the ‘benefit-sharing loophole’ emerges through the acknowledgement that the parties to the Nagoya Protocol are able to include reference to dematerialized pathogen samples within their national ABS legislation, requiring a user to obtain PIC and to negotiate MAT which define conditions for the access and utilization of DSI, as well as the sharing of benefits arising from its use.

Under the Nagoya Protocol, each Party is obliged to take ‘appropriate, effective and proportionate legislative, administrative or policy measures’—including the implementation of national ABS legislation^87^—‘to provide that genetic resources utilized within its jurisdiction have been accessed in accordance with prior informed consent and that mutually agreed terms have been established’.^88^ The flexibility afforded to the Parties of the Nagoya Protocol here has made it possible for ‘genetic resources’ to be expanded in accordance with the individual requirements of each Party, with it being possible to capture DSI and the benefits resulting from its use within the ABS transaction as a result.^89^ This suggested avenue for closing the benefit-sharing loophole is not purely hypothetical in nature, with a growing number of countries having already taken steps to regulate DSI within their national ABS legislation or within the terms and conditions associated with access to physical samples. In 2015, for example, new ABS legislation was adopted in Brazil which governed the utilization of information surrounding the ‘genetic origin of plant, animal or microbial species’.^90^ The consequence here is the closing of the benefit-sharing loophole, albeit on a national level, which reduces the threat posed by the dematerialization of pathogen samples to equity, fairness, and global health justice.

However, the flexibility afforded to the Parties to the Nagoya Protocol in relation to the implementation of national ABS legislation will produce significant differences across states. As the WHO has acknowledged, this will function to produce a ‘patchwork of different legislation and measures covering the sharing of DSI’, which would produce greater problems as opposed to providing a true solution and closing the ‘benefit-sharing loophole’.^91^ This ‘patchwork’ of legislation is likely to impose ABS obligations which range from stifling to non-existent, with the result here being an increase in ‘jurisdiction shopping’ by those seeking to obtain access to DSI, with the intention of finding the least restrictive benefit-sharing obligations.^92^ Rather than ensuring the fair and equitable distribution of benefits resulting from the use of DSI and thereby closing the ‘benefit-sharing loophole’, the reliance on national ABS legislation and the risk of ‘jurisdiction shopping’ here functions to further limit the distribution of benefits during a health emergency to those states with the least demanding obligations, if benefit-sharing occurs at all.

### IV.B. Including DSI within the Scope of the CBD, the Nagoya Protocol and the PIP Framework

Perhaps the most obvious solution to the ‘benefit-sharing loophole’ and the threat pathogen dematerialization poses to global health justice would be to expand the definition of genetic resources under the CBD and the Nagoya Protocol, alongside the definition of PIP BM under the PIP Framework, to include DSI, thereby capturing it within the respective ABS transactions. Current lines of argument, however, suggest that it is not necessary to expand these definitions as both already use appropriate terms that are sufficient to include reference to DSI.^93^ This is based on the understanding that the use of the ‘material’ within the definition of both genetic resources and PIP BM is broad enough to include reference to information.^94^ Taking this approach to closing the ‘benefit-sharing loophole’ would seemingly introduce greater consistency, whilst ensuring that DSI is subject to benefit-sharing obligations. Contention exists, however, within the international community as to whether the definition of genetic resources or PIP BM could or should be recognized as including DSI.^95^

According to Bagley, the main views that exist surrounding DSI and the scope of the CBD/Nagoya Protocol can be split into three, with the first being that ‘DSI is not within the definition of “genetic resources” but may result from utilization of genetic resources and can be addressed in MAT. Beyond MAT, DSI itself is a global non-monetary benefit and no further benefit-sharing for its use need be provided’.^96^ This is typically the view of powerful developed states and non-state actors who are strong advocates for DSI remaining beyond the requirements of the ABS transaction in order to avoid additional benefit-sharing obligations,^97^ producing considerable tension between the Global North and the Global South as a result. During COP13, for example, whilst Canada and other members of the Global North were astute in their belief that ‘the Nagoya Protocol is about the ABS of genetic material only’, as opposed to genetic resources and DSI, many delegations from the Global South were keen to stress the fact that ‘resources are precious due to information that they contain’.^98^

The second view is that ‘“genetic resources” should be interpreted to include DSI such that DSI is subject to PIC/MAT under the Nagoya Protocol’, although a combination of unrealistic expectations and impractical solutions with regards to how DSI could be governed under the current ABS regime have underscored a lack of support for this approach during discussions.^99^ The final view for consideration here is that ‘DSI is not within the definition of “genetic resources,” but does result from their utilization. Monetary benefits should be shared from commercial uses’ but that ‘non-monetary benefits, while important, are not sufficient to comply with Nagoya Protocol obligations’.^100^ With this seemingly being the prevailing viewpoint, going so far as to somewhat bridge the gap between the Global North and Global South, it remains unlikely—but not impossible—that the definition of ‘genetic resources’ under the CBD/Nagoya Protocol with be interpreted to include DSI in the future.

Similar discussions have occurred within the context of the PIP Framework, although the leaving view here is that the definition of PIP BM should not be expanded to include DSI as it should not be ‘subject to the liabilities associated with the legally binding SMTA2 requirements’.^101^

The lack of consensus—alongside the apparent power imbalance, which exists here between the Global North and the Global South—suggest that neither the interpretation of the pre-existing definitions of ‘genetic resources’ and ‘PIP BM’ as including DSI, nor the expansion of those definitions, is probable, despite the existence of ‘credible suggestions’ that the current definitions are poorly suited to the problems which the use of ABS aims to overcome.^102^

### IV.C. A New Multilateral ABS Framework

Despite this, there have recently been some developments with regards to DSI and the CBD specifically which offer support for a greater willingness amongst the Global North to tackle the growing problems caused by DSI with regards to ABS. Whilst DSI in the context of both the CBD and the Nagoya Protocol has been the subject of discussions and negotiations for over five years,^103^ following COP14 and the adoption of Decision 14/20^104^ there has been greater focus on overcoming the divergent views amongst Parties with regards to the place of DSI under the Convention. Whilst the inclusion of DSI within the definition of ‘genetic resources’ under the CBD and Nagoya Protocol is still unlikely, broad support now exists for the creation of a multilateral benefit-sharing system where access to DSI would be granted in exchange for monetary benefit-sharing via an international fund.^105^ Despite this, diverging viewpoints and delay caused by the COVID-19 pandemic leave both the form such a system would take and how it would work in practice unresolved to date, with it being unclear just what—if any—impact this would have on pathogen sharing and the delivery of justice for developing states during a health emergency.

An alternative solution would be for the WHO to design a new multilateral ABS framework which is specific to pathogen sharing and explicitly includes DSI within its scope.^106^ It would be vital that the new framework capture DSI within the ABS transaction, closing the ‘benefit-sharing loophole’ and ensuring the distribution of subsequent benefits in such a way that ensures fairness, equity, and justice in global public health.^107^ It is plausible that the development new multilateral ABS Framework could be influenced the PIP Framework, through the use of SMTAs for example, although there remains the risk that using the Framework as a model or source of guidance may present ‘significant implementational and operational challenges’.^108^ Whilst the adoption of a new multilateral ABS Framework here would arguably succeed in closing the ‘benefit-sharing loophole’ whilst ensuring the fair and equitable distribution of benefits, due to the ability to make it ‘fit for purpose’, it is questionable as to whether a new multilateral ABS instrument which includes DSI within its scope would gain sufficient support from non-state actors or whether the best result would simply be another non-binding resolution.^109^ Regardless of the form which a new ABS Framework would take, it is important to note that, as it would operate alongside rather than instead of the Nagoya Protocol, it would need to be recognized within the national ABS legislation of all states in order to succeed; a feat which may prove difficult to achieve if support for a new Framework is lacking.

With the Member States of the WHO currently negotiating the ‘Pandemic Treaty’ and the working draft text recognizing that ‘equity should remain as a principle, an indicator and an outcome of pandemic prevention, preparedness and response’,^110^ a new multilateral framework containing reference to ABS is likely to emerge in the future; although the Treaty is not expected to stray far from the current ABS regime. Current drafts of the Treaty continue to favor the use of the ABS transaction as a means of achieving equity, going beyond the scope of the PIP Framework to include as a minimum ‘rapid, regular and timely sharing of pathogens and genomic sequences through a standardized real-time global platform; and timely access to affordable, safe and effective pandemic response products, including diagnostics, vaccines, personal protective equipment and therapeutics’.^111^ With the Pandemic Treaty expected to develop a ‘comprehensive system for access and benefit sharing’ which will ensure the sharing of both physical pathogen samples and DSI ‘through one or more standardized real-time platforms available to all Parties’,^112^ clear reference to DSI exists, although it remains unclear as to whether this reference will extend to the inclusion of DSI in relation to benefit-sharing obligations under the Treaty or whether these will remain restricted to physical pathogen samples and the benefits resulting from their use.

### IV.D. Potential Problems With Including DSI Within ABS

Whilst each of the solutions that have been considered by this paper fail to provide a viable solution to the threat posed by pathogen dematerialization to ABS and global health justice for one reason or another, their ultimate flaw is their determination to fix the problem by including DSI within the ABS transaction. The inclusion of DSI within the ABS transaction is neither advisable nor well-suited, with two significant problems being likely to emerge should such inclusion ever occur.

#### i. Problems with monitoring and tracing

Should DSI ever be included within the ABS transaction, difficulties will emerge in relation to the ability to monitor and track its use;^113^ with the intangible nature of DSI facilitating its rapid transfer and the ease with which it is modified in practice.^114^ Although monitoring DSI is considered to be a ‘theoretical possibility’, a suitable method has yet to be identified,^115^ with there remaining the risk that no mechanism for the monitoring and tracking of DSI would be able to accurately identify everyone who has accessed and utilized it.^116^ This raises particular issues in the context of the ABS transaction as fairness, equity, and justice would require that all third-party users of DSI be subject to benefit-sharing obligations under the ABS transaction, not simply those which are capable of being traced.^117^ Further issues arise in relation to the fact that not all circumstances where DSI is accessed or viewed are equal, with it being possible to access DSI for a number of purposes which vary considerably in their value, ranging from viewing it out of interest but not using it to accessing it in order to develop medical countermeasures.^118^ ABS obligations in relation to DSI would therefore need to acknowledge these differences and distinguish between them accordingly in order to truly reflect fairness and equity but this would not always be possible as third-party users may not be aware of how they intend to use DSI at the time which it is accessed.^119^ Whilst a notice at the point of access detailing information around benefit-sharing expectations has been proposed as a possible solution here,^120^ it is questionable as to how effective this would be in practice.

#### ii. The risk to scientific research

Although the inclusion of DSI within the ABS transaction may go some way to closing the ‘benefit-sharing loophole’ and reducing the threat posed to global health justice, there remains the risk that it will actually limit the fair and equitable distribution of benefits as a result of it stifling necessary and timely scientific advancements.^121^ Many within the scientific community have expressed concern that open research and development—both of which are vital for pandemic preparedness—would be significantly hampered should DSI be subject to ABS obligations under the CBD, the Nagoya Protocol, the PIP Framework, or a new multilateral ABS framework.^122^ This is a direct result of the complex and burdensome nature of ABS and the likely delays and restrictions on scientific research which are expected to emerge should DSI be included within the ABS transaction. Whilst the ability of developing states to access benefits is undoubtedly threatened by the ability of DSI to bypass the ABS transaction, delay in scientific advancements, in the development of medical countermeasures during a health emergency, and so on would pose an equal threat; suggesting that more needs to be done to balance the interests of all relevant stakeholders, instead of simply expanding the scope of the CBD, the Nagoya Protocol and the PIP Framework or creating a new framework.

### IV.E. ABS & DSI: A Poor Fit

There is little doubt that the ABS transaction is an unsatisfactory means of ensuring fair and equitable access to the benefits resulting from the use of DSI.^123^ Afterall, ABS and DSI appear to be entirely at odds with one another, with the sharing of DSI finding its basis in open access and free exchange whilst the ABS transaction produces restrictions, obligations, and burdensome complexities.^124^ None of the possible solutions to the ‘benefit-sharing loophole’ considered within this paper are viable or capable of delivering equity and justice, with this ultimately raising the question of whether ABS is truly the only means of ensuring both access to DSI and the fair and equitable distribution of the benefits resulting from its use.^125^ An alternative solution must be found which adequately balances the need for open and timely access to DSI and for fair and equitable benefit-sharing; something which the ABS transaction is unable to do. In the face of rapid scientific and technological advances, alongside the growing role of DSI within global public health, a solution must be found which closes the ‘benefit-sharing loophole’ whilst ensuring that fairness, equity and justice are upheld.

## V. CONCLUDING REMARKS: THE NEED FOR AN ALTERNATIVE SOLUTION

In order to ensure the realization of fairness, equity, and justice in global public health, DSI must ultimately not be allowed to bypass the ABS obligations contained within the CBD, the Nagoya Protocol, the PIP Framework, or any other ABS mechanisms which may emerge in the future. However, the current suggestions for how to capture DSI within the ABS transaction are insufficient, often producing greater problems in the long run.

As has been evidenced within this paper, the ABS transaction is a ‘poor policy fit’^126^ for ensuring both access to DSI and the fair and equitable sharing of the benefits resulting from its use. As such, it is it is vital that greater consideration is afforded to the question of whether there is an alternative means of achieving the aims of the ABS transaction—namely the equitable distribution of benefits arising from the utilization of genetic resources or access to PIP BM—in the context of access to DSI, without actually capturing DSI within the ABS transaction.^127^

The way forward here may be to take a step away from the use of ABS, not just as a potential solution to the benefit-sharing loophole and the growing threat of pathogen dematerialization, but in the regulation of pathogen sharing overall. After all, there have long been questions surrounding the suitability of ABS to pathogens,^128^ with this seemingly being the case regardless of whether they are in their physical form or dematerialized. The challenge, therefore, is to provide a more suitable mechanism—or mechanisms—for resolving the two issues acknowledged within the Introduction: the pressing need for researchers and other actors within the field of pandemic preparedness to gain access to novel pathogen samples in a timely manner; and the limited availability of vaccine and other medical countermeasures during a health emergency, with developing states typically struggling to gain sufficient access to such resources in a timely manner due to developed states dominating procurement.^129^ Now is the time to finally treat these issues for what they are—separate, distinct and unsuited to a single solution—whilst still recognizing that alternative solutions must be found simultaneously due to the perceived dependency of developing states on their ability to trade their sovereign genetic resources in order to access vaccine and other medical countermeasures during a health emergency. Whilst problems are likely to emerge in the search for alternative solutions, particularly with regards to the difficulties faced by developing states in procuring vaccine and other medical countermeasures during a health emergency, delinking the two issues is vital in order to truly ensure fairness, equity and justice in global public health.^130^

## Footnotes

2 Una Jakob, *Norm Conflicts in Global Health: The Case of Indonesia and Pandemic Influenza Preparedness,* PRIF Working Paper No. 47, https://www.ssoar.info/ssoar/bitstream/handle/document/69029/ssoar-2020-jakob-Norm_Conflicts_in_Global_Health.pdf?sequence=1&isAllowed=y&lnkname=ssoar-2020-jakob-Norm_Conflicts_in_Global_Health.pdf 4 (2020); Arnold S. Monto, *Reflections on The Global Influenza Surveillance and Response System (GISRS) at 65 Years: An Expanding Framework for Influenza Detection, Prevention and Control*, 12 Influenza Other Respi Viruses 10, 10 (2018).

3
*Id.*

4 Mark Eccleston-Turner and Michelle Rourke, *Arguments Against the Inequitable Distribution of Vaccines Using the Access And Benefit Sharing Transaction*, 70 International and Comparative Law Quarterly 825, 826 (2021).

5 Michelle Rourke, *The History of Accessing and Sharing Human Pathogens for Public Health Research* in Viral Sovereignty and Technology Transfer the Changing Global System for Sharing Pathogens for Public Health Research 31 (Sam Halabi and Rebecca Katz ed., 2020).

6 Eccleston-Turner and Rourke, *supra* note 4, at 826.

7 The Indonesian Government cited Article 15 of the Convention of Biological Diversity which recognises ‘the sovereign rights of States over their natural resources’ and their subsequent authority to determine if and how those resources are accessed.

8 The recognition of pathogens as ‘genetic resources’ under the Convention on Biological Diversity has been the subject of much debate as a result of the belief that they run contrary to the Convention’s aims and represent ‘only a threat’. Despite this, considerable support exists with regards to the recognition of pathogen samples as ‘genetic resources’ as a result of their wide-ranging value and use, alongside the recognition by the WHO during the 2007 H5N1 influenza pandemic that the Convention was something which could apply to H5N1 influenza samples. Whilst such recognition may still be a point of contention, pathogens are now broadly understood to fall within the scope of ‘genetic resources’ and will be considered as such for the purpose of this paper. See: International Chamber of Commerce, *Pathogens and the International Regime on Access and Benefit-Sharing*, Policy Brief Document No. 450/1051, Paris: International Chamber of Commerce, https://www.cbd.int/abs/submissions/abswg-08-icc-en.pdf (Sep. 11, 2009); Morten Walløe Tvedt, *Beyond Nagoya: towards a legally functional system of access and benefit sharing* in Global Governance of Genetic Resources Access and Benefit Sharing After the Nagoya Protocol (Sebastian Oberthür and G. Kristin Rosendal ed., 2014).

9 Michael A. Stevenson and Andrew F. Cooper, *Overcoming Constraints of State Sovereignty: global health governance in Asia*, 30(7) Third World Quarterly 1379, 1380 (2009); Jakob, *supra* note 2, at 7.

10 Simone Vezzani, *Preliminary Remarks on the Envisaged World Health Organization Pandemic Influenza Preparedness Framework for the Sharing of Viruses and Access to Vaccines and Other Benefits*, 13(6) World Intellectual Property 675, 677 (2010).

11 Eccleston-Turner and Rourke, *supra* note 4, at 829.

12 See: See: Mark Eccleston-Turner, *Operationalizing the Right to Health through the Pandemic Influenza Preparedness Framework*, XII(1) Global Health Governance, 22–33 (2018).

13 Developed states dominated vaccine procurement throughout the COVID-19 pandemic. By March 2021, they had pre-ordered approximately 70% of the global supply of COVID-19 vaccine for the year. This supply of vaccine would be used for around 16% of the global population. See: Oliver J Wouters, Kenneth C Shadlen, Maximilian Salcher-Konrad, Andrew J Pollard, Heidi J Larson, Yot Teerawattananon et al, *Challenges in ensuring global access to COVID-19 vaccines: production, affordability, allocation, and deployment,* 397(10278) The Lancet, 1023–1034 (2021).

14 This paper utilises several phrases, such as biological materials, physical samples, physical pathogen samples and physical genetic resources, which should all be understood to refer back to ‘genetic resources.’ Differences in the language utilised here are a result of the need to draw a distinction between ‘genetic resources’ as a whole and (physical) pathogen samples for the purpose of this paper and wider discussion. Equally, variations in the terminology used here, particularly with regards to direct quotations which this paper utilises, serve to reflect the inconsistencies in language which are present throughout the literature and policy documents, highlighting the level of uncertainty which exists not only around DSI but around genetic resources under the ABS regime as a whole.

15 Eccleston-Turner and Rourke, *supra* note 4, at 828.

16 Whilst ‘synthetic biology’ lacks universal definition, this paper utilises the operational definition of the Ad Hoc Technical Expert Group (AHTEG) on Synthetic Biology: ‘synthetic biology’ is ‘a further development and new dimension of modern biotechnology that combines science, technology and engineering to facilitate and accelerate the understanding, design, redesign, manufacture and/or modification of genetic materials, living organisms and biological systems,’ with the process of pathogen dematerialisation offering a clear example of this. See: Convention on Biological Diversity, *REPORT OF THE AD HOC TECHNICAL EXPERT GROUP ON SYNTHETIC BIOLOGY *Montreal, Canada (2015) UNEP/CBD/SYNBIO/AHTEG/2015/1/3 https://www.cbd.int/doc/meetings/synbio/synbioahteg-2015-01/official/synbioahteg-2015-01-03-en.pdf?dowload

17 Rourke, *supra *note 5, at 31 and 37.

18 Whilst the ABS regime has inadvertently captured pathogens within its scope as a result of the actions of Indonesia in 2007, it is important to recognise that it was formed within the biodiversity regime and expanded to capture traditional knowledge as a form of restitution and justice to community groups. For more on the history of ABS within biodiversity and the role which it has played with regards to traditional knowledge and the provision of justice to community groups, please see: Carmen Richerzhagen, *Effective Governance of Access and Benefit-Sharing under the Convention on Biological Diversity,* 20 Biodiversity and Conservation 2243, 2243 (2011); Vandana Shiva, the Violence of the Green Revolution: Third World Agriculture, Ecology, and Politics 257 (1991); Evanson C. Kamauand Gerd Winter (ed.) Genetic Resources, Traditional Knowledge and the Law (2009); Debra Harry, *Biocolonialism and Indigenous Knowledge in United Nations Discourse,* 20(3) Griffith Law Review, 702–728 (2011).

19 Eccleston-Turner and Rourke, *supra* note 4, at 829.

20 Charles Lawson and Michelle Rourke, *Digital sequence information as a marine genetic resource under the proposed UNCLOS legally binding instrument,* 122(103878) Marine Policy 1, 1 (2020).

21 Convention on Biological Diversity (CBD) (adopted June 5, 1992, entered into force Dec. 29, 1993) 1760 UNTS 79.

22 Nagoya Protocol on Access to Genetic Resources and the Fair and Equitable Sharing of Benefits Arising from their Utilization to the Convention on Biological Diversity (Nagoya Protocol) (adopted Oct. 29, 2010, entered into force Oct. 12, 2014) UNEP/CBD/COP/DEC/X/1.

23 Pandemic Influenza Preparedness Framework for the sharing of influenza viruses and access to vaccines and other benefits (PIP Framework) (adopted May 24, 2011; Second edition, 2021) A64/VR/10.

24 Although there are a number of international ABS instruments which are aligned with the CBD, such as the International Treaty on Plant Genetic Resources for Food and Agriculture (ITPGRFA), as the focus of this paper is solely on the sharing of pathogen samples, only the CBD, the Nagoya Protocol and the PIP Framework will be considered here.

25 CBD, *supra* note 21, Article 1. The objective of the Nagoya Protocol expands upon the foundational objective of the CBD to include ‘the fair and equitable sharing of the benefits arising from the utilization of genetic resources, including by appropriate access to genetic resources and by appropriate transfer of relevant technologies, taking into account all rights over those resources and to technologies, and by appropriate funding, thereby contributing to the conservation of biological diversity and the sustainable use of its components.’ See: Nagoya Protocol, *supra *note 22, Article 1.

26 See: CBD, *supra* note 21, Article 15.4 and 15.5.

27 Nagoya Protocol, *supra* note 22, Article 6.

28 Linda Wallbott, Franziska Wolff and Justyna Pożarowska, *The negotiations of the Nagoya Protocol Issues, coalitions and process* in Global Governance Of Genetic Resources Access and Benefit Sharing After the Nagoya Protocol 35 (Sebastian Oberthür and G. Kristin Rosendal ed., 2014).

29 Nagoya Protocol, *supra *note 22, Article 5.

30 Wallbott, Wolff and Pożarowska, *supra *note 28, at 35.

31 See: Maria Julia Marinissen, Ruvani Chandrasekera, John Simpson, Theodore Kuschak, and Lauren Barna, *Sharing of Biological Samples during Public Health Emergencies Challenges and Opportunities for National and International Action* in Viral Sovereignty and Technology Transfer the Changing Global System for Sharing Pathogens for Public Health Research (Sam Halabi and Rebecca Katz ed., 2020).

32 PIP Framework, *supra* note 23, Article 2.

33
*Id.* Article 3.

34 Eccleston-Turner and Rourke, *supra* note 4, at 827.

35
*Id*.

36 PIP Framework, *supra* note 23, Article 5(1).GISRS refers to the Global Influenza Surveillance and Response System. It is a network of public health laboratories which operates on an international scale and co-ordinates the sharing of influenza virus samples. For more on this, see: World Health Organisation, *Global Influenza Surveillance and Response System (GISRS)*  https://www.who.int/influenza/gisrs_laboratory/en/

37 PIP Framework, *supra* note 23, Article 6(11).

38 Nicole Jefferies, *Levelling the playing field? Sharing of influenza viruses and access to vaccines and other benefits,* 20 JLM 59, 66 (2012).

39
*Id.* at 64.

40
*Id.* at 66.

41 PIP Framework, *supra *note 23, Annex 2. These benefits include, but are not limited to, the donation of vaccine and the donation of antiviral medication.

42 Jefferies, *supra* note 38, at 66.

43 As of December 2022, only Seqirus UK Limited have committed to the donation of 10% of their real time pandemic vaccine production to WHO during the next pandemic. The result here is a much lower supply of donated vaccine in the virtual stockpile than was originally intended by the Framework, with this ultimately reducing potential vaccination coverage in developing states during the next pandemic. See: World Health Organisation, *Category A: Summary of Signed Agreements and Benefits*  https://cdn.who.int/media/docs/default-source/pip-framework/smta2/smta2-cata-25aug2020.pdf?sfvrsn=d004e66_2.

44 Eccleston-Turner and Rourke, *supra* note 4, at 828.

45
*Id.* at 839.

46 See: R Chennells, *Toward Global Justice through Benefit- Sharing*, 40 Hastings Center Report 3 (2010); D Schroeder, *Justice and Benefit Sharing* in Indigenous Peoples, Consent and Benefit Sharing (R Wynberg, D Schroeder and R Chennells ed., 2009); Mzm Nomani, *The Access and Benefit-Sharing Regime: An Environmental Justice Perspective*, 49 Environmental Policy and Law, 259–263 (2020).

47 David P. Fidler and Lawrence O. Gostin, *The WHO Pandemic Influenza Preparedness Framework: A Milestone in Global Governance for Health, *306(2) Jama 200, 201 (2011).

48 ‘Biopiracy’ is ‘the unidirectional transfer or appropriation of the genetic resources and associated knowledge of indigenous and local communities, with little or no regard to knowledge holders.’ See: Chidi Oguamanam, *Genetic Resources & Access and Benefit Sharing: Politics, Prospects and Opportunities for Canada after Nagoya*, 22(2) Journal of Environmental Law and Practice 87–201, 93 (2011).

49 ‘Bio-colonialism’ is ‘the collection and appropriation of cultural and natural heritage into museums, zoological and botanical gardens and other ex situ collections in colonizing countries.’ See: Elisa Morgera, Elsa Tsioumani, and Matthias Buck, Unraveling the Nagoya Protocol a Commentary on the Nagoya Protocol on Access and Benefit-Sharing to the Convention on Biological Diversity 7 (2014).

50 For more on the development of ABS as a means of rectifying colonial inequalities, see: Sebastian Oberthür and G. Kristin Rosendal, *Global governance of genetic resources: background and analytical framework* in Global Governance of Genetic Resources Access and Benefit Sharing After the Nagoya Protocol (Sebastian Oberthür and G. Kristin Rosendal ed., 2014); Linda Wallbott, Franziska Wolff and Justyna Pożarowska, *The negotiations of the Nagoya Protocol Issues, coalitions and process* in Global Governance of Genetic Resources Access and Benefit Sharing After the Nagoya Protocol (Sebastian Oberthür and G. Kristin Rosendal ed., 2014).

51 Makarim Wibisono, *Responsible Virus Sharing and Benefit Sharing: A Balance between Humankind and the Pandemic Threat* in Pandemic Preparedness in Asia 124 (Mely Caballero-Anthony ed., 2009).

52 Eccleston-Turner and Rourke, *supra* note 4, at 828.

53 For an overview of the general benefits which can be derived from synthetic biology in global public health, see: Chidi Oguamanam, *ABS: Big Data, Data Sovereignty and Digitization: A New Indigenous Research Landscape *in Genetic Resources, Justice and Reconciliation: Canada and Global Access and Benefit Sharing (Chidi Oguamanam ed., 2018) https://ssrn.com/abstract=3326282 or http://dx.doi.org/10.2139/ssrn.3326282

54 As has been noted above, dematerialisation here refers to the act(s) of separating information from physical pathogen samples. This act(s) reduces the need of pharmaceutical companies and research institutes to access physical samples.

55 In discussions surrounding the PIP Framework, this information is referred to as Genetic Sequence Data (GSD). Whilst it is important to recognise the differences in the terminology utilised during discussion surrounding DSI or GSD across difference regimes, this paper will treat the two as interchangeable terms and will favour the use of ‘DSI’ throughout.

56 DSI in these discussions refers to ‘both indicative and contextual information including nucleic acid sequence reads and the associated data, information about the sequence assembly, annotation and maps describing whole genomes, individual genes or fragments, barcodes, organelle genomes and single nucleotide polymorphisms, information about gene expression, data on macromolecules and cellular metabolites, information on ecological relationships and abiotic factors of the environment, and so on.’ See: Charles Lawson and Michelle Rourke, *Digital sequence information as a marine genetic resource under the proposed UNCLOS legally binding instrument, *122(103878) Marine Policy 1, 2 (2020).

57 World Health Organisation, *New Technologies Using Genetic Sequence Data Fact Sheet* (2018) https://cdn.who.int/media/docs/default-source/pip-framework/governance/analysis-of-seasonal-influenza-gsd-under-the-pip-framework/analysis-document/newtech_en_3apr2018-2.pdf?sfvrsn=2cf27757_5

58 Michelle F. Rourke, Alexandra Phelan and Charles Lawson, *Access and benefit-sharing following the synthesis of horsepox virus,* 38 Nat Biotechnol 537, 538 (2020).

59 Molly R. Bond and Deborah Scott, *Digital biopiracy and the (dis)assembling of the Nagoya Protocol,* 117 Geoforum 24, 26 (2020).

60 Rourke, Phelan and Lawson, *supra* note 58, at 538.

61 World Health Organisation, *supra* note 57.

62 Jakob, *supra* note 2, at 3; Fran Humphries, Michelle Rourke, Todd Berry, Elizabeth Englezos, and Charles Lawson, *COVID-19 Tests the Limits of Biodiversity Laws in a Health Crisis: Rethinking “Country of Origin” for Virus Access and Benefit-sharing,* 28 Journal of Law and Medicine 684, 688 (2021).

63 Eccleston-Turner and Rourke, *supra* note 4, at 841.

64 Michael Heinrich et al, *Access and Benefit Sharing Under the Nagoya Protocol—Quo Vadis? Six Latin American Case Studies Assessing Opportunities and Risk,* 11(765) Front. Pharmacol. 1, 15 (2020).

65 Bond and Scott, *supra* note 59, at 24.

66 Charles Lawson, Michelle Rourke and Fran Humphries, *Information as the latest site of conflict in the ongoing contests about access to and sharing the benefits from exploiting genetic resources, *10(1) Queen Mary Journal of Intellectual Property 7, 19 (2020).

67 Bond and Scott, *supra* note 59, at 26.

68
*Id. *

69
*Id.* at 24.

70 Michelle Rourke, Mark Eccleston-Turner, Alexandra Phelan and Lawrence Gostin, *Policy opportunities to enhance sharing for pandemic research, *368(6492) Science 716, 717 (2020).

71 CBD, *supra* note 21, Article 2.

72 Rourke, Phelan and Lawson, *supra* note 58, at 538.

73 Bond and Scott, *supra* note 59, at 27.

74 Rourke, Phelan and Lawson, *supra* note 58, at 538.

75 PIP Framework, *supra* note 23, Article 4.1.

76 Lawson and Rourke, *supra* note 20, at 1. General obligations relating to the sharing of information can be found in Article 17 of the CBD and Article 14 of the Nagoya Protocol; whilst the sharing of ‘information’ is referenced in various contexts throughout the PIP Framework.

77
*Id. *

78
*Id. *

79 World Health Organisation, *Approaches to Seasonal Influenza and Genetic Sequence Data Under the PIP Framework* 18 (Dec. 14, 2018) www.who.int/influenza/pip/WHA70108b_Analysis.pdf.

80 Rourke, Phelan and Lawson, *supra* note 58, at 538.

81
*Id.* at 537.

82 Humphries, Rourke, Berry, Englezos, and Lawson, *supra* note 62, at 693.

83 Bond and Scott, *supra* note 59, at 31.

84 Jakob, *supra* note 2, at 17.

85 Bond and Scott, *supra* note 59, at 31.

86 Rourke, Eccleston-Turner, Phelan and Gostin, *supra* note 70, at 717.

87 Kanchana Kariyawasam & Matthew Tsai, *Access to genetic resources and benefit sharing: Implications of Nagoya Protocol on providers and users,* 21 The Journal of World Intellectual Property 289, 290 (2018).

88 Nagoya Protocol, *supra* note 22, Article 15.

89 Charles Lawson, Fran Humphries and Michelle Rourke, *The future of information under the CBD, Nagoya Protocol, Plant Treaty, and PIP Framework,* 22 The Journal of World Intellectual Property 103, 107 (2019).

90 Bruce S. Manheim, *Regulation of synthetic biology under the Nagoya Protocol,* 34(11) Nature Biotechnology 1104, 1105 (2016).

91 WHO, *supra* note 79, at 21.

92
*Id. *

93 Lawson and Rourke, *supra* note 20, at 5.

94
*Id. *

95 Joost Flach, Carolina dos S. Ribeiro, Mark B. van der Waal, Raymond X. van der Waal, Eric Claassen and Linda H.M. van de Burgwal, *The Nagoya Protocol on Access to Genetic Resources and Benefit Sharing: Best practices for users of Lactic Acid Bacteria*, 9 Pharmanutrition 1, 2 (2019).

96 Margo A. Bagley, *“Just” Sharing: The Virtues of Digital Sequence Information Benefit-Sharing for the Common Good,* 63(1) Harvard International Law Journal 1, 29 (2022).

97 Lawson and Rourke, *supra *note 20, at 2.

98 Bond and Scott, *supra *note 59, at 27.

99 Bagley, *supra* note 96, at 34.

100
*Id.* at 41.

101 WHO, *The Inclusion of Seasonal Influenza Viruses and Genetic Sequence Data (GSD) in the Context of the Pandemic Influenza Preparedness (PIP) Framework A position paper from the Directors of the GISRS WHO Collaborating Centers and Essential Regulatory Laboratories of GISRS* 4 (https://cdn.who.int/media/docs/default-source/pip-framework/governance/analysis-of-seasonal-influenza-gsd-under-the-pip-framework/submissions-related-to-implementation-of-wha70(10)8(b)/cc_erl_directorspositionpaper_8b.pdf?sfvrsn=94969d24_5&ua=1)

102 Lawson, Humphries and Rourke, *supra *note 89, at 107.

103 Convention on Biological Diversity, ‘Digital sequence information on genetic resources’ Intersessional Period 2017–2018 https://www.cbd.int/doc/notifications/2017/ntf-2017-037-abs-en.pdf

104 Convention on Biological Diversity, ‘DECISION ADOPTED BY THE CONFERENCE OF THE PARTIES TO THE CONVENTION ON BIOLOGICAL DIVERSITY 14/20. Digital sequence information on genetic resources’ Sharm El-Sheikh, Egypt, Nov. 17–29, 2018 CBD/COP/DEC/14/20 https://www.cbd.int/doc/decisions/cop-14/cop-14-dec-20-en.pdf

105 Convention on Biological Diversity, ‘Digital sequence information on genetic resources Draft decision submitted by the President’ Montreal, Canada, Dec. 7–19, 2022 CBD/COP/15/L.30 https://www.cbd.int/doc/c/c181/12cf/d29ef8c3f6bd4ec701699d9d/cop-15-l-30-en.pdf

106 Rourke, Eccleston-Turner, Phelan and Gostin, *supra* note 70, at 718.

107 Bond and Scott, *supra* note 59, at 31.

108 Eccleston-Turner and Rourke, *supra* note 4, at 854.

109 Rourke, Eccleston-Turner, Phelan and Gostin, *supra* note 70, at 718.

110 World Health Organisation, Working draft, presented on the basis of progress achieved, for the consideration of the Intergovernmental Negotiating Body at its second meeting, July 13, 2022, A/INB/2/3, 3.

111
*Id.* at 10.

112
*Id.* at 11.

113 Lawson, Humphries and Rourke, *supra* note 89, at 113.

114 World Health Organisation, *supra* note 79, at 25.

115 Lawson, Humphries and Rourke, *supra* note 89, at 114.

116 World Health Organisation, (n 82), *supra* note 79, at 25.

117
*Id. *

118
*Id.*

119
*Id.*

120
*Id.*

121 Lawson and Rourke, *supra *note 20, at 7. See also: Jorge L. Contreas, *COVID-19 as an Example of Why Genomic Sequence Data Should Remain Patent Ineligible* in COVID-19 Policy Playbook: Legal Recommendations for a Safer, More Equitable Future. Boston: Public Health Law Watch (Scott Burris, Sarah de Guia, Lance Gable, Donna Levi, Wendy E. Parmet and Nicolas Terry ed., 2021)

122 Sarah Laird, Rachel Wynberg, Michelle Rourke, Fran Humphries, Manuel Ruiz Muller, and Charles Lawson, *Rethink the expansion of access and benefit sharing, *367(6483) Science 1200–1202 (2020).

123
*Id.* at 1200.

124
*Id.*

125
*Id.*

126
*Id.*

127 Rourke, Phelan and Lawson, *supra* note 58, at 538.

128 See: Meeting of the Pandemic Influenza Preparedness Advisory Group, Oct. 17–19, 2018. Report to the Director-General’ Geneva: World Health Organization (2018) https://www.who.int/publications/m/item/meeting-of-the-pandemic-influenza-preparedness-framework-advisory-group-17-19-october-2018; Claire Lajaunie and Serge Morand, *Nagoya Protocol and Infectious Diseases: Hindrance or Opportunity?*, 8 Frontiers in Public Health 1–4 (2020); Carolina dos S Ribeiro, Marion P. Koopmans and George B. Haringhuizen, *Threats to timely sharing of pathogen sequence data,* 362(6413) Science 404–406 (2018).

129 Eccleston-Turner and Rourke, *supra* note 4, at 829.

130
*Id*. at 856–858.

